# Rab14 Regulates Maturation of Macrophage Phagosomes Containing the Fungal Pathogen Candida albicans and Outcome of the Host-Pathogen Interaction

**DOI:** 10.1128/IAI.02917-14

**Published:** 2015-03-17

**Authors:** Blessing Okai, Natalie Lyall, Neil A. R. Gow, Judith M. Bain, Lars-Peter Erwig

**Affiliations:** Aberdeen Fungal Group, University of Aberdeen, Aberdeen, United Kingdom

## Abstract

Avoidance of innate immune defense is an important mechanism contributing to the pathogenicity of microorganisms. The fungal pathogen Candida albicans undergoes morphogenetic switching from the yeast to the filamentous hyphal form following phagocytosis by macrophages, facilitating its escape from the phagosome, which can result in host cell lysis. We show that the intracellular host trafficking GTPase Rab14 plays an important role in protecting macrophages from lysis mediated by C. albicans hyphae. Live-cell imaging of macrophages expressing green fluorescent protein (GFP)-tagged Rab14 or dominant negative Rab14, or with small interfering RNA (siRNA)-mediated knockdown of Rab14, revealed the temporal dynamics of this protein and its influence on the maturation of macrophage phagosomes following the engulfment of C. albicans cells. Phagosomes containing live C. albicans cells became transiently Rab14 positive within 2 min following engulfment. The duration of Rab14 retention on phagosomes was prolonged for hyphal cargo and was directly proportional to hyphal length. Interference with endogenous Rab14 did not affect the migration of macrophages toward C. albicans cells, the rate of engulfment, the overall uptake of fungal cells, or early phagosome processing. However, Rab14 depletion delayed the acquisition of the late phagosome maturation markers LAMP1 and lysosomal cathepsin, indicating delayed formation of a fully bioactive lysosome. This was associated with a significant increase in the level of macrophage killing by C. albicans. Therefore, Rab14 activity promotes phagosome maturation during C. albicans infection but is dysregulated on the phagosome in the presence of the invasive hyphal form, which favors fungal survival and escape.

## INTRODUCTION

Candida albicans is a major fungal pathogen of humans that lives within the normal mucosal flora of the gastrointestinal tract in about 80% of healthy adults but can be pathogenic when host defenses are compromised (1). Each year, C. albicans and other Candida species cause more than 75 million vaginal infections in women and 400,000 systemic infections in immunocompromised individuals (2). Systemic infection is associated with mortality rates of >30% even with pharmacological intervention (3).

Host defense against candidiasis relies mainly on the ingestion and elimination of fungal cells by phagocytes of the innate immune system (4). Following internalization, pathogens are confined in phagosomes, which are vacuoles derived from the plasma membrane. These phagosomes undergo extensive remodelling, termed phagosomal maturation, by acquiring microbicidal and lytic enzymes delivered by membrane fusion and fission events with different endolysosomal compartments (5). These events lead to the progressive acidification of the phagosome lumen, the acquisition of a full arsenal of antimicrobial features, including the activation of hydrolytic enzymes, and ultimately, the formation of the microbicidal phagolysosome (6). Most pathogens are killed and degraded in mature phagolysosomes, but some can escape or subvert the phagosome maturation process; these include Mycobacterium tuberculosis, Listeria monocytogenes, Coxiella burnetii, Brucella species, Salmonella enterica serovar Typhimurium, Helicobacter pylori, Shigella flexneri, Chlamydia species, Legionella pneumophila, Francisella tularensis, Rhodococcus equi, Leishmania donovani, Trypanosoma species, Toxoplasma gondii, Histoplasma capsulatum, Candida glabrata, and C. albicans (7–18).

Rab proteins are central regulators of the dynamic processes of phagosome maturation (5). The composition of Rab GTPases localized in the phagosome membrane defines the biochemical composition and intracellular behavior of the phagosome, determining fusion partners and defining the lipid composition of the membrane (19). Rab GTPases therefore regulate vesicle recruitment and the modulation of vesicular transport through interactions with cytoskeletal components (20). Phagosome purification combined with proteomics approaches have identified several dozen Rab GTPases that associate with phagosomes (21–23). Of these, Rab5 and Rab7 are the best characterized with regard to phagosome maturation. Rab5 associates rapidly and transiently with phagosomes following phagocytosis and is essential for the fusion of early endosomes with phagosomes (24). Rab7 has been shown in a number of studies to associate with the phagosomal membrane and plays a key role in mediating interactions with late endocytic/lysosomal compartments (25, 26). Although the functions of many phagosomal Rab proteins have been well characterized, only a few of the >60 Rabs identified have been investigated with regard to their function in phagosome maturation.

Rab14 is an important protein that regulates the interaction of phagosomes with early endocytic compartments, but its role in the maturation of phagosomes containing fungal cells has not been examined. This GTPase has been found to localize to the Golgi and rough endoplasmic reticulum compartments and to early endosomes (27). Proteomics studies have revealed that Rab14 associates with phagosomes containing latex beads (21), and studies performed with the slime mold Dictyostelium suggest that a Rab14-related GTPase localizes in the endolysosomal pathway and regulates phagosome-lysosome fusion (28). In macrophages infected with M. tuberculosis, Rab14 is actively recruited to phagosomes containing these bacteria, and this association disrupts phagosome maturation (29). Likewise, host Rab14 is required for the intracellular growth of S. enterica (30).

Here we have combined live-cell imaging with genetic manipulation of host macrophages to study the dynamic role of Rab14 in phagosome maturation during infection by C. albicans. We show that Rab14 localizes transiently to phagosomes containing live C. albicans cells shortly after engulfment. In contrast to the transient association of Rab5 with phagosomes, the prolonged retention of Rab14 on phagosomes was dependent on fungal morphology and proportional to hyphal length. Manipulation of Rab14 by small interfering RNA (siRNA) or expression of dominant negative variants had no effect on markers of early phagosome maturation but delayed the acquisition of key markers of late stages of the maturation process. Importantly, a consequence of interference with Rab14 was a significant increase in the ability of the pathogen to escape from and kill macrophages after phagocytosis. Therefore, we show that Rab14 plays an important role in protecting macrophages against killing by C. albicans and that engulfment of the invasive hyphal form of the fungus alters Rab14 dynamics, delaying phagolysosome formation.

## MATERIALS AND METHODS

### Culture of macrophage cell lines.

The RAW 264.7 and J774.1 murine macrophage cell lines (ECACC, HPA, Salisbury, United Kingdom) were cultured every 4 to 5 days in Dulbecco's modified Eagle medium (DMEM) (Lonza, Slough, United Kingdom) supplemented with 10% (vol/vol) heat-inactivated fetal calf serum (FCS) (Biosera, Ringmer, United Kingdom), 200 U/ml penicillin-streptomycin (Invitrogen, Paisley, United Kingdom), and 2 mM l-glutamine (Invitrogen) at 37°C under 5% CO_2_.

### Culture of mouse BMDM.

Work was approved by the Animals Scientific Procedures Division, Home Office, United Kingdom Government, under the personal license of Lars-Peter Erwig (PIL60/6194) and project license PPL60/4007, in line with the European Union Directive 2010/63/EU on the Protection of Animals Used for Scientific Purposes. Experiments were approved by the College of Life Sciences and Medicine Ethics Review Board, University of Aberdeen, with adherence to local and institutional policy requirements.

Intact femurs and tibiae were aseptically removed from the hind legs of C57BL/6 mice obtained from the Brown group, University of Aberdeen. Bone marrow cells were extracted using a 25-gauge needle and were passed through an 18-gauge needle to obtain a homogenous mixture. Cells were plated in petri dishes in bone marrow medium: Iscove's modified Dulbecco's medium (IMDM) (Gibco) supplemented with 30% L929 conditioned medium, 10% FCS, 1× nonessential amino acids (Gibco), and 100 U penicillin–0.1 mg/ml streptomycin at 37°C in a 5% CO_2_ incubator. Fresh medium was added to cells on day 3, and on day 7, bone marrow-derived macrophages (BMDM) were detached from culture vessels by gentle scraping for subsequent use in phagocytosis experiments.

### Culture of C. albicans.

Cultures of C. albicans serotype A strain CAI4+CIp10 (NGY152) and the Δ*efg1*/Δ*efg1* strain (CA79) obtained from glycerol stocks frozen at −80°C were maintained on solid synthetic complete medium without uracil (SC-Ura medium) prepared from 0.67% (wt/vol) yeast nitrogen base without amino acids (Formedium, Norfolk, United Kingdom), 2% (wt/vol) technical agar (Oxoid, Cambridge, United Kingdom), 1 mM NaOH solution (BDH Chemicals, VWR International, Leicestershire, United Kingdom), double-distilled H_2_O, 0.1% (wt/vol) adenine hemisulfate (Sigma-Aldrich, Dorset, United Kingdom), 4% (wt/vol) glucose (Fisher Scientific, Leicestershire, United Kingdom), and 0.4% SC-Ura dropout mixture (Formedium). A single colony of C. albicans from the plate was cultured in 5 ml of SC-Ura liquid medium (the same recipe as above, without the agar) at 30°C overnight with shaking at 200 rpm.

### Transfection of macrophages.

The *eGFP-Rab14*, *eGFP-Rab14*^*S25N*^, and *eGFP-Rab14*^*N124I*^ plasmid constructs were kindly provided by Cecile Itzstein (Bone and Musculoskeletal Research Programme, University of Aberdeen) and have been described previously (27). The *eGFP-Rab5* and *tRFP-Rab7* plasmids were constructed as described previously (18). Murine Rab5 cDNA was amplified from a plasmid (OriGene, Rockville, MD, USA) by using a proofreading polymerase (KOD; Merck Millipore, Darmstadt, Germany) with primers 5′-GCCGCCGAATTCCCATGGCTAATCGA-3′ and 5′-GAGCGGCCGGGATCCTAGTTACTACA-3′ for Rab5 (Eurofins Genomics, Ebersberg, Germany) to generate EcoRI and BamHI sites (Roche, Welwyn Garden City, United Kingdom) for cloning into pEGFP-C3 (Clontech, Mountain View, CA, USA). Murine Rab7 cDNA was digested from a plasmid (OriGene) with SgfI and MluI (Roche) and was inserted into pCMV-AN-tRFP (OriGene). Sequences were verified (DNA Sequencing Services, University of Dundee, Dundee, United Kingdom) prior to transfection.

For plasmid DNA transfections, RAW 264.7 macrophages were seeded onto Iwaki glass-based dishes (VWR, Leicestershire, United Kingdom) in antibiotic-free complete DMEM at a density of 6 × 10^5^ cells per dish and were incubated for 24 h until 80% confluence was reached. The macrophages were transfected with 19 μl Lipofectamine LTX and 6 μl Plus reagent (Invitrogen, Carlsbad, CA) with 3 μg of plasmid DNA according to the manufacturer's instructions before being replenished with fresh medium and then incubated for a further 15 to 21 h.

For siRNA transfections, 1 × 10^6^ J774.1 or RAW264.7 macrophages or BMDM were seeded onto imaging dishes or standard 6-well culture plates for RNA or protein extraction in antibiotic-free DMEM and were incubated overnight. Cells were transfected with Lipofectamine 2000, Opti-MEM (Invitrogen, United Kingdom), and 25 nM Silencer Select Rab14 siRNA (Invitrogen, United Kingdom) for 24 h. The effects of Rab14 siRNA were compared with those of a nontargeting negative-control siRNA and a transfection reagent only.

For siRNA and plasmid DNA cotransfections, RAW 264.7 macrophages that had been seeded in antibiotic-free DMEM on the previous day were transfected with siRNA on day 1. Plasmid DNA was transfected on day 2. Macrophages were transfected with siRNA 48 h prior to imaging, which yielded satisfactory knockdown (see Fig. S4 in the supplemental material).

### RNA isolation and real-time PCR (RT-PCR).

Total RNA was first isolated from macrophages using a standard TRIzol (Invitrogen) protocol and then purified using an RNeasy kit (Qiagen) according to the manufacturer's protocol. mRNA was converted to cDNA with random primers (Promega), SuperScript reverse transcriptase, and oligo(dNTP) nucleotides (Invitrogen) according to the manufacturer's instructions. As a negative control, a reaction mixture without the addition of reverse transcriptase was included.

To detect Rab14 expression, 50 ng of cDNA was combined with a TaqMan Gene Expression primer/probe assay for Rab14 with a 6-carboxyfluorescein (FAM) probe (Applied Biosystems). A VIC probe-based glyceraldehyde-3-phosphate dehydrogenase (GAPDH) assay (Applied Biosystems) was run in parallel as an internal control. RT-PCR was performed in a LightCycler 480 system (Roche, Mannheim, Germany) detecting FAM (emission wavelength, 520 nm) and VIC (emission wavelength, 555 nm) from amplification events of Rab14 and GAPDH, respectively. Data were analyzed with LightCycler 480 software, version 1.5.0.

### Western blot analysis.

Whole-cell lysates were prepared from macrophages transfected with control or Rab14 siRNA by using radioimmunoprecipitation assay (RIPA) lysis buffer containing 1% Triton X-100, 0.5% sodium deoxycholate, and a protease inhibitor cocktail (Roche) in phosphate-buffered saline (PBS). Protein concentrations were determined by a Bradford assay, and 20 μg per well was first loaded and separated on a NuPAGE Novex 4–12% Bis-Tris gel (Invitrogen) and then transferred to a nitrocellulose membrane. The membrane was blocked for 1 h in Tris-buffered saline (TBS)–Tween (TBST) buffer (0.1%) containing 5% (wt/vol) skim milk powder at room temperature. Overnight, the membrane was probed with a mouse anti-Rab14 antibody (Santa Cruz Biotechnology) at 1 μg ml^−1^ in blocking buffer. Following three washes with TBST, the blot was probed with a horseradish peroxidase (HRP)-conjugated goat anti-mouse secondary antibody (Santa Cruz Biotechnology) at 0.2 μg ml^−1^ for 1 h at room temperature and finally was subjected to enhanced chemiluminescence detection. Equal loading of protein was confirmed by probing the same blot with a monoclonal anti-β-actin antibody (Sigma-Aldrich).

### Live-cell video microscopy phagocytosis assays.

Live-cell video microscopy phagocytosis assays were carried out at 37°C using a DeltaVision Core microscope (Applied Precision, Issaquah, WA, USA) or an UltraVIEW VoX spinning disk microscope (Nikon, Surrey, United Kingdom). Volocity software was used for data analysis (Improvision, PerkinElmer, Coventry, United Kingdom). Immediately prior to live-cell imaging, DMEM was replaced with 2 ml prewarmed supplemented CO_2_-independent medium (Gibco, Invitrogen, Paisley, United Kingdom).

A live C. albicans cell suspension was added to macrophages at a multiplicity of infection of 3:1, and macrophage acidic compartments were stained with 1 mM LysoTracker Red DND-99 (LTR) (Invitrogen). Volocity software (version 6.3.1; Improvision) was set to capture images every 1 min for a 6-h period using an electron-multiplying charge-coupled device (EMCCD) camera. For all conditions, at least three independent experiments were carried out, with a minimum of five movies per experiment. Fifty to 100 macrophages were selected randomly from each experiment for analysis of migration, engulfment, and marker localization.

### Survival of C. albicans following phagocytosis.

Standard phagocytosis assays were conducted as described above. Following 4 h (when macrophage lysis is detectable, hyphal filaments are not too long, and microbial degradation may have occurred), the survival of fungi was determined by harvesting cells and lysing macrophages by resuspension in H_2_O. C. albicans was plated from serial dilutions onto solid SC-Ura medium, and the viability of fungi was determined from the number of CFU.

### Immunofluorescence and confocal microscopy.

Macrophages were fixed with 2% paraformaldehyde for 5 min, permeabilized with saponin for 25 min, blocked with 5% normal goat serum for 1 h, and stained for Rab14 using anti-Rab14 antibody produced in rabbit (Sigma-Aldrich) at 10 μg ml^−1^. An Alexa Fluor 555-conjugated goat anti-rabbit secondary antibody at 10 μg ml^−1^ was incubated for 1 h. Rab14 staining was performed on untransfected cells (RAW264.7 macrophages and BMDM) and RAW264.7 macrophages expressing green fluorescent protein (GFP)-labeled Rab14, with or without phagocytosis of C. albicans. To stain LAMP1-positive phagosomes, J774.1 macrophages were prepared for phagocytosis assays with C. albicans. The macrophages were washed with PBS to remove unbound C. albicans cells and were fixed with 4% paraformaldehyde for 15 min, followed by a blocking step with blocking buffer (10% skim milk powder, 0.3% Tween 20 in PBS) for 45 min. The cells were then incubated with an allophycocyanin (APC)-conjugated antibody against mouse LAMP1 (10 μg ml^−1^; BioLegend, London, United Kingdom) for 1 h at room temperature. After washing, cells were visualized under a Zeiss 510 Meta laser scanning confocal microscope with a 40× oil immersion objective.

### Cathepsin B activity assay.

The bioactivity of secreted cathepsin B was detected using the Cathepsin B Activity Assay kit (ImmunoChemistry, Inc.). This fluorescence-based assay utilizes the cathepsin B substrate arginine-arginine (RR) coupled with cresyl violet (Magic Red). Samples containing cathepsin enzymes will cleave at one or both arginine (R) amide linkage sites, releasing the cresyl violet fluorophore (excitation wavelength, 550 to 590 nm). The signal will intensify as the red fluorescent product accumulates in the lysosome. J774.1 macrophages plated on imaging dishes were preincubated with live C. albicans cells at a 3:1 ratio for 1 h in supplemented CO_2_-independent medium. At the end of the incubation period, the fluorogenic cathepsin B substrate cresyl violet (ImmunoChemistry, Inc.) was added to the cells, and live imaging of the last 1 h of the interaction was performed.

### Statistical analysis.

Experimental data were analyzed using the GraphPad Prism statistical analysis package (GraphPad Software, Inc., USA), and results are expressed as average values ± standard errors of the means (SEM). The Mann-Whitney test and one-way analysis of variance (ANOVA), followed by Bonferroni *post hoc* tests, were used to determine statistical significance.

## RESULTS

### Dysregulation of Rab14 association with phagosomes containing C. albicans cells.

**FIG 1:**
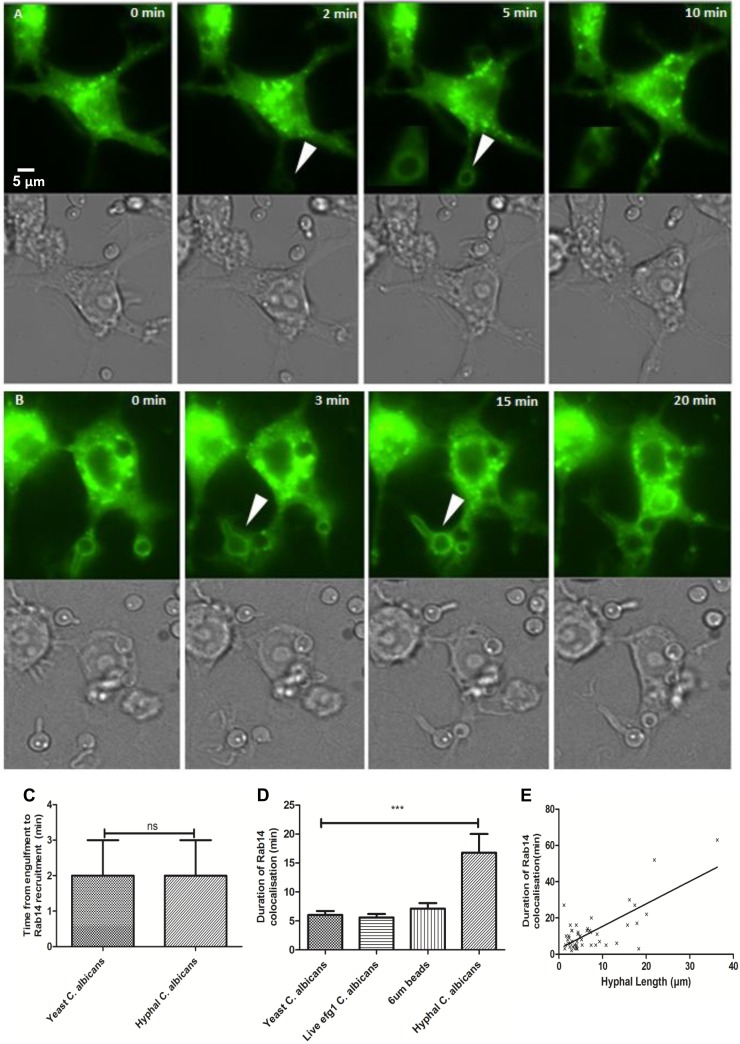
Rab14 associates transiently and differentially with phagosomes containing C. albicans yeast or hyphal cells. (A and B) Live RAW 264.7 macrophages were transfected with eGFP-Rab14, allowed to ingest C. albicans cells, and monitored by live-cell imaging using fluorescence (upper panels) and differential interfer ence contrast microscopy (lower panels). Bar, 5 μm. The corresponding movies can be viewed in Videos S1 and S2 in the supplemental material. (A) RAW 264.7 macrophages were transfected with eGFP-Rab14 for 16 h, treated with live C. albicans cells, and imaged using live-cell microscopy over a 6-h period. Selected frames depict events from the time of contact between a live C. albicans yeast cell and the macrophage to the time of Rab14 localization. The white arrows indicate colocalization between phagosomes containing C. albicans cells and Rab14. (B) Selected frames from a live-cell microscopy movie showing detailed events from the time of contact between a live C. albicans hyphal cell and the macrophage to the time of Rab14 localization. (C) Time from the engulfment of C. albicans yeast or hyphal cells to the start of Rab14 recruitment. A Mann-Whitney statistical test was performed because the data were skewed; ns, no significant difference. (D) Duration of Rab14 localization on phagosomes containing either yeast or hyphal cells. ANOVA and *post hoc* Bonferroni tests were performed to determine significant differences (***, *P* ≤ 0.001). (E) Positive correlation (Spearman's) between hyphal length and the duration of Rab14 retention on phagosomes containing C. albicans hyphal cells. Data are means ± SEM for at least three independent experiments.

In order to visualize the temporal phagosomal localization of Rab14, RAW 264.7 macrophages were transfected to express enhanced green fluorescent protein (eGFP)-tagged Rab14 (Fig. 1). Live-cell video microscopy enabled detailed analysis of the temporal localization of Rab14 to phagosomes containing live C. albicans cells. Prior to the onset of phagocytosis, clusters of Rab14 vesicles were localized diffusely around the cell, concentrated around the perinuclear region, as shown previously for other cell types (27). A commercially available anti-Rab14 antibody was used to confirm the binding pattern of Rab14 identified by eGFP-Rab14-expressing macrophages (see Fig. S1 in the supplemental material). Following the internalization of C. albicans cells by macrophages, newly formed phagosomes transiently acquired Rab14 approximately 2 min after the completion of engulfment. Rab14 remained associated with phagosomes for an average of 6.0 ± 0.7, 5.6 ± 0.6, or 6.8 ± 1.0 min on phagosomes containing the WT strain (CAI4-CIp10) ingested in yeast form, a yeast-locked mutant (*efg1*Δ) strain, or 6-μm latex beads, respectively (Fig. 1). The presence of Rab14 on phagosomes containing C. albicans cells was confirmed by staining of primary cells (BMDM), which are not amenable to transfection, with an anti-Rab14 antibody (see Fig. S2 in the supplemental material). Next, we examined whether the phagosomal localization of Rab14 was influenced by the morphology of the ingested fungal particle. Live phagocytosis imaging permits the observation of Rab14 dynamics on individual phagosomes containing yeast-locked fungi, yeast cells that subsequently germinate into hyphae, and fungi that have already germinated prior to engulfment. As with phagosomes containing the yeast form of C. albicans, localization of Rab14 to phagosomes containing hyphae occurred within 2 min (Fig. 1). Interestingly, phagosomes containing hyphal fungal cells retained Rab14 for 16.8 ± 3.2 min—around 3-fold the duration seen on phagosomes containing yeast cells (Fig. 1D). Hyphal lengths were measured using a line tool (Volocity; PerkinElmer), and the correlation of the hyphal length at the time of engulfment with the duration of the retention of Rab14 on phagosomes was determined. A positive correlation was observed (*R_s_* = 0.35) between hyphal length and the duration of Rab14 recruitment to phagosomes (Fig. 1E). Figure 1A and B show a time lapse sequence of images in macrophages transfected with eGFP-Rab14 and infected with live C. albicans cells in either the yeast or the hyphal form. (The corresponding movies can be viewed in Videos S1 and S2 in the supplemental material, respectively).

**FIG 2:**
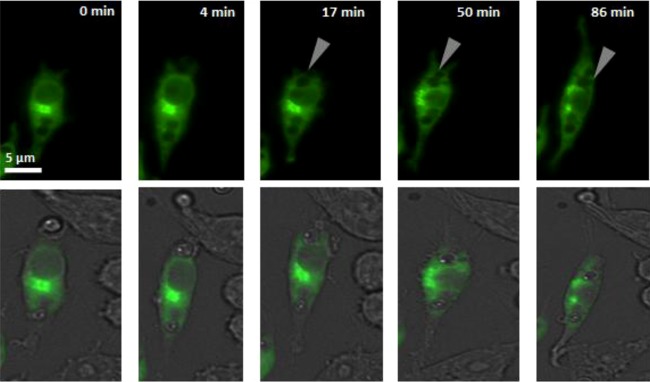
eGFP-Rab14^S25N^ is not localized to phagosomes following C. albicans uptake. (Top) Selected frames from a live-cell movie showing RAW 264.7 macrophages transfected with eGFP-Rab14^S25N^. The time elapsed is given at the top. The gray arrows indicate phagosomes containing C. albicans cells that fail to exhibit localization of eGFP-Rab14^S25N^. (Bottom) Differential interference contrast/green merge images. At least 30 macrophages were examined per experiment. Three independent experiments were carried out. The corresponding movie can be viewed in Video S3 in the supplemental material.

Additional control experiments used RAW264.7 macrophages transfected with dominant negative controls (eGFP-Rab14^S25N^ [locked in an inactive GDP-bound state] and eGFP-Rab14^N124I^ [unable to bind GDP/GTP, and therefore inactive]). Rab14 did not localize to phagosomes following phagocytosis of live C. albicans cells in the dominant negative control macrophages, even after extended periods (Fig. 2; see also Video S3 in the supplemental material), demonstrating that Rab14 was required to be in its active GTP-bound state in order to be localized to phagosomes containing live C. albicans cells. These data suggest that active Rab14 localizes to phagosomes containing C. albicans cells within 2 min following engulfment and that the initial localization is independent of the morphology of the fungal cell. However, the duration of Rab14 retention was significantly longer after the engulfment of hyphae than after that of yeast cells and was proportional to hyphal size.

### Depletion of endogenous Rab14 does not affect uptake.

To investigate the effect of inhibiting Rab14 on phagosome maturation and the ultimate outcome of the host-pathogen interaction, it was important to first confirm that the methods employed did not affect the migration of macrophages toward C. albicans cells, the rate of engulfment, or the overall uptake of C. albicans cells by macrophages.

Two methods were used to interfere with Rab14 function in macrophages: (i) siRNA-mediated knockdown of Rab14 expression in J774.1 macrophages and BMDM and (ii) transfection of RAW 264.7 macrophages with eGFP-tagged constructs to express dominant negative variants eGFP-Rab14^S25N^ and eGFP-Rab14^N124I^. First, we addressed the question of whether macrophage migration toward C. albicans cells was affected by Rab14 depletion. siRNA-mediated knockdown reduced endogenous Rab14 expression by as much as 90% (see Fig. S3 in the supplemental material). Macrophage migration was assessed over a 6-h period in our standard live-cell phagocytosis assay by tracking individual cells using Volocity imaging software, version 6.3.1 (Improvision, PerkinElmer), as in our previous studies (31, 32).

**FIG 3:**
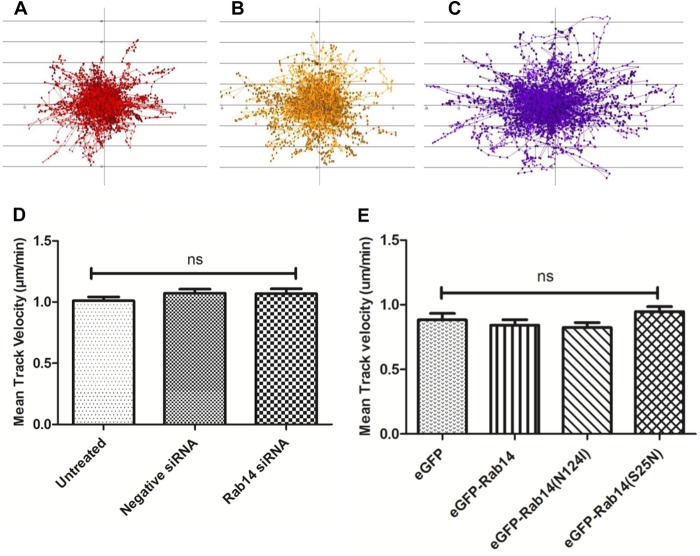
Knockdown of Rab14 by siRNA does not affect the migration of macrophages toward C. albicans cells. (A to C) Tracking diagrams illustrate the distances traveled and the directionality and velocity of J774.1 macrophages that had been left untreated (A) or transfected with negative-control (B) or Rab14 (C) siRNA in response to live C. albicans cells. Tracks represent the movements of individual phagocytes relative to their starting positions; symbols mark the locations of phagocytes at 1-min intervals; and arrows indicate directionality. (D and E) Depletion of Rab14 either by siRNA-mediated knockdown (D) or by the expression of dominant negative Rab14 variants (E) does not affect the mean track velocity of macrophages in response to C. albicans cells. Data are means ± SEM for at least three independent experiments. ANOVA and Bonferroni *post hoc* tests were carried out. ns, no significant difference.

**FIG 4:**
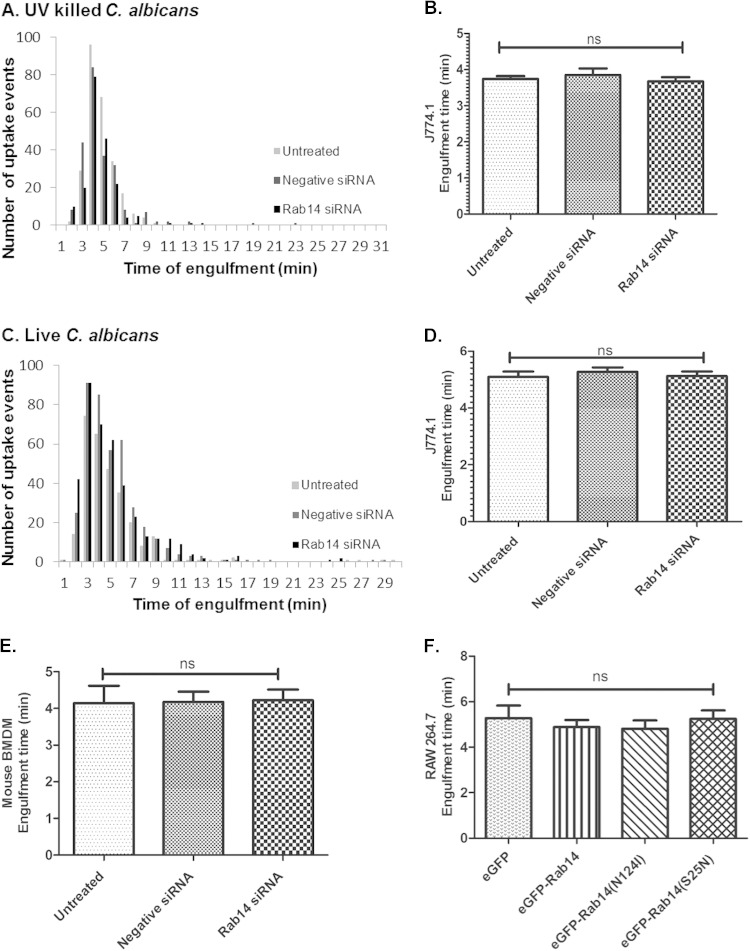
The rate of engulfment of UV-killed or live C. albicans cells is not affected by Rab14 knockdown. (A to D) Frequency of uptake and time taken for untreated, negative-control siRNA-treated, or Rab14 siRNA-treated J774.1 macrophages to fully engulf UV-killed (A and B) or live (C and D) C. albicans cells. (E) Average time taken for untransfected, negative-control siRNA-transfected, or Rab14 siRNA-transfected BMDM to ingest live C. albicans cells. (F) Average time taken for RAW 264.7 macrophages transfected with eGFP, eGFP-Rab14, eGFP-Rab14^N124I^, or eGFP-Rab14^S25N^ to ingest live C. albicans cells. Data are means ± SEM for at least three independent experiments. ANOVA and Bonferroni *post hoc* tests were carried out. ns, no significant difference.

Directional movement and velocity in response to live C. albicans cells did not differ between untreated macrophages, macrophages transfected with negative-control siRNA, and macrophages with siRNA-mediated Rab14 knockdown (Fig. 3A to C). Mean track velocities were not significantly different for untreated (1.01 ± 0.03 μm/min), negative-control siRNA-transfected (1.07 ± 0.03 μm/min), and Rab14 siRNA-transfected (1.07 ± 0.03 μm/min) macrophages (Fig. 3D). Similar results were also found in comparisons between macrophages transfected to express eGFP-tagged Rab14, eGFP-Rab14^S25N^, and eGFP-Rab14^N124I^ (Fig. 3E). Next, we investigated whether Rab14 depletion affected the overall rate of engulfment of C. albicans cells by macrophages following cell-cell contact. The average times to engulfment (from the first cell-cell contact to complete internalization) of UV-killed C. albicans cells by untreated, negative-control siRNA-transfected, and Rab14 siRNA-transfected macrophages were not significantly different (3.7 ± 0.1 min, 3.9 ± 0.2 min, and 3.7 ± 0.1 min, respectively) (Fig. 4A and B). In keeping with previous observations, the times to engulfment of live C. albicans cells were longer than those for UV-killed cells (28) but, again, not significantly different for untreated, negative-control siRNA-transfected, and Rab14 siRNA-transfected macrophages. (5.1 ± 0.2 min, 5.3 ± 0.2 min, and 5.1 ± 0.2 min, respectively) (Fig. 4C and D). We also observed a similar trend in the engulfment kinetics of BMDMs and of macrophages transfected with *eGFP-Rab14* and dominant negative variants (Fig. 4E and F). Therefore, Rab14 does not accelerate or delay macrophage migration toward fungal cells or the rate of their engulfment.

### Rab14 depletion does not interfere with early phagosome maturation.

**FIG 5:**
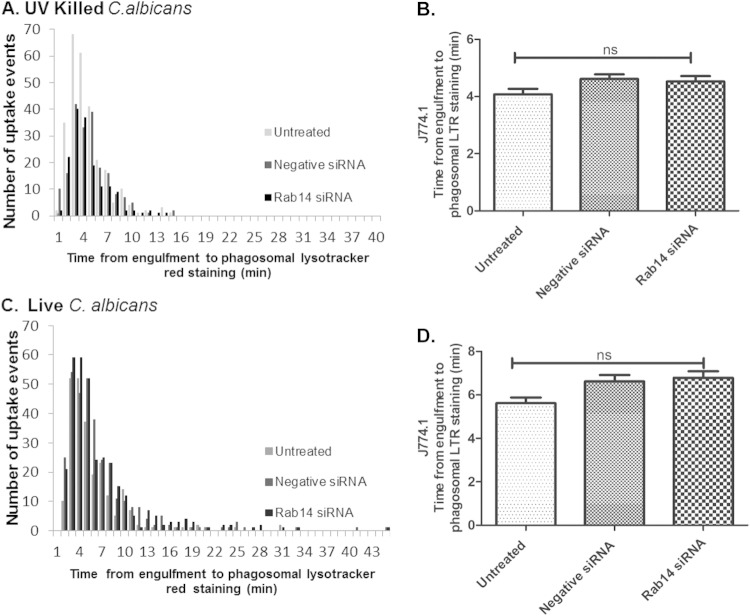
The localization of LysoTracker Red (LTR) to phagosomes containing C. albicans cells is unaffected in Rab14-depleted macrophages. J774.1 macrophages were either left untransfected or transfected with negative-control siRNA or Rab14 siRNA prior to incubation with UV-killed (A and B) or live (C and D) C. albicans cells. (A and C) Numbers of uptake events for macrophages at different time intervals. (B and D) Average times taken for LTR localization to phagosomes, determined by analysis of movies generated from live imaging at 1-min intervals. ANOVA and Bonferroni *post hoc* tests were carried out. ns, no significant difference.

The localization of Rab14 to fungal phagosomes shortly after engulfment, shown above, suggested that Rab14 may interfere with early phagosome maturation. LysoTracker Red (LTR) is highly selective for acidic organelles and was used here to measure early phagosomal acidification by live-cell video microscopy. The average times between engulfment and the localization of LTR to phagosomes containing either live or UV-killed C. albicans cells were similar for untreated, negative-control siRNA-transfected, and Rab14 siRNA-transfected macrophages. The average times taken for LTR localization to phagosomes containing UV-killed C. albicans cells were 4.1 ± 0.2 min, 4.6 ± 0.2 min, and 4.5 ± 0.2 min for untreated, negative-control siRNA-treated, and Rab14 siRNA-treated macrophages, respectively (Fig. 5A and B). A similar pattern was found for phagosomes containing live C. albicans cells: LTR localization took 5.6 ± 0.3 min, 6.1 ± 0.2 min, and 6.1 ± 0.2 min for untreated, negative-control siRNA-treated, and Rab14 siRNA-treated macrophages, respectively (Fig. 5C and D). Thus, Rab14 depletion did not affect early acidification dynamics as measured by LTR labeling.

**FIG 6:**
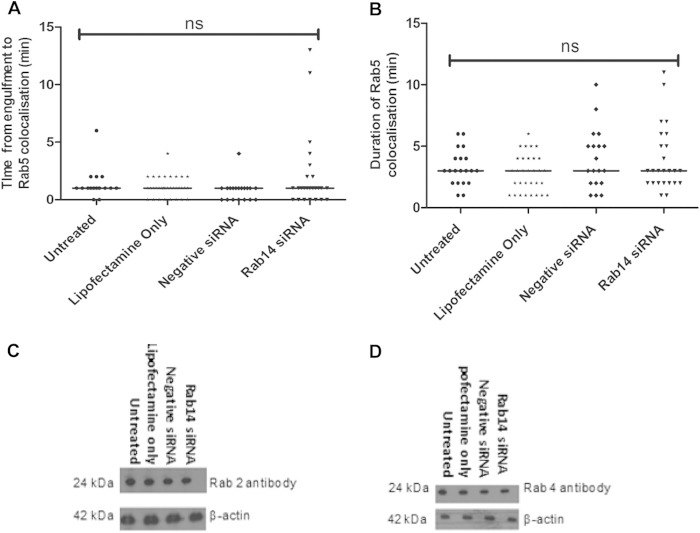
The localization of eGFP-Rab5 to phagosomes containing C. albicans cells is unaffected in Rab14-depleted macrophages. (A) Time taken for Rab5 to be recruited to phagosomes following the engulfment of live C. albicans cells by siRNA-transfected RAW 264.7 macrophages. (B) Duration of Rab5 recruitment to RAW 264.7 macrophages. (A and B) Symbols indicate phagosomes analyzed from at least three independent experiments, with means shown. ANOVA and Bonferroni *post hoc* tests were carried out. ns, no significant difference. (C and D) J774.1 macrophages transfected with siRNA to knock down Rab14 were lysed, and the protein expression levels of Rab2 (C) and Rab4 (D) were determined by Western blot analysis.

Rab14 associates with phagosomes transiently, and we hypothesize that this association may be important in the regulation of phagolysosome biogenesis. To further understand the role of Rab14, we investigated whether markers of early and late stages of the phagosome maturation process were affected in macrophages with reduced Rab14 expression. RAW 264.7 macrophages depleted of Rab14 (using siRNA) were cotransfected with *eGFP-Rab5* and were infected with live C. albicans cells. Western blot and quantitative PCR (qPCR) analyses were performed to confirm that Rab14 knockdown was maintained in the context of eGFP-Rab5 expression (see Fig. S4 in the supplemental material). Live imaging revealed that Rab5 associated transiently with phagosomes containing C. albicans cells as early as <1 min after uptake and that depletion of Rab14 did not affect the kinetics of this recruitment (Fig. 6A). Rab5 dissociated from phagosomes after 3 min, on average, in control and Rab14 siRNA-treated macrophages (Fig. 6B), suggesting that Rab5 localization to phagosomes containing C. albicans cells occurs prior to Rab14 localization and that the retention kinetics of Rab5 is independent of Rab14.

Next, we examined whether Rab14 depletion affected the expression of Rab GTPases Rab2 and Rab4, which share high sequence similarity and are regulatory proteins associated with early endosomes (33, 34). The expression of Rab2 and Rab4 was determined by Western blot analysis. Even when Rab14 was depleted by 90%, no difference was detected in the protein level of either Rab2 or Rab4 (Fig. 6C and D).

### Rab14 depletion does not interfere with phagosomal acquisition of Rab7.

**FIG 7:**
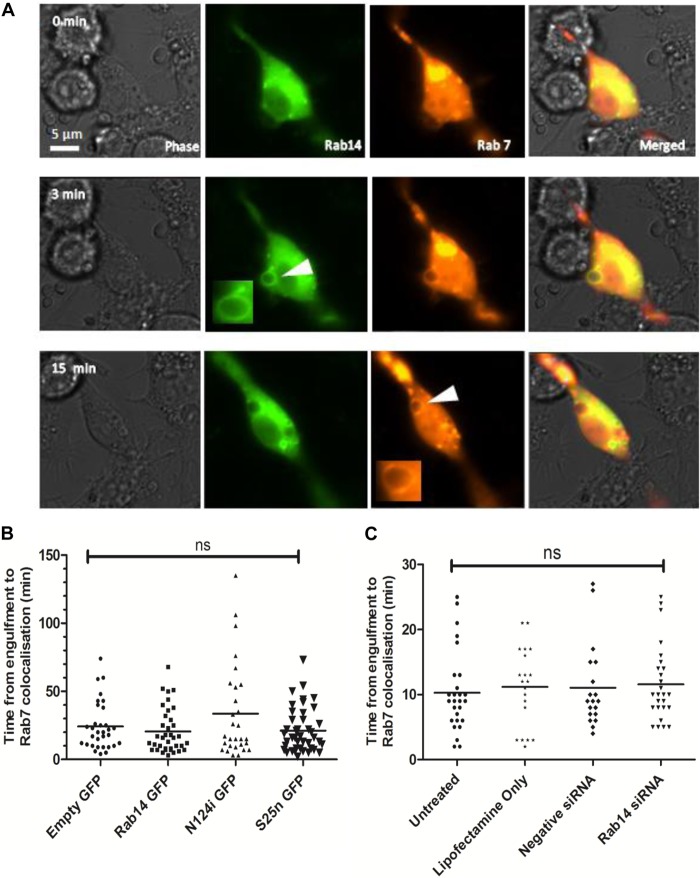
Rab7 localization is unaffected by Rab14 depletion. (A) Time lapse images showing the recruitment of Rab14 and Rab7 to phagosomes containing C. albicans cells. RAW 264.7 cells were cotransfected with eGFP-Rab14 (green) and tRFP-Rab7 (red), incubated with C. albicans cells, and monitored by live-cell imaging with differential interference contrast and fluorescence microscopy. The association of Rab14 with phagosomes (arrowhead in central panel) was followed by Rab7 accumulation (arrowhead in bottom panel). Similar results were obtained from cotransfected macrophages in four independent experiments. The corresponding movie can be viewed in Video S4 in the supplemental material. (B) Time to recruitment of tRFP-Rab7 to phagosomes containing live C. albicans cells. RAW 264.7 macrophages were cotransfected with tRFP-Rab7 and wild-type or dominant negative Rab14. (C) RAW264.7 macrophages were either left untreated or transfected with either Lipofectamine only, negative-control siRNA, or Rab14 siRNA. Symbols indicate phagosomes measured during at least three independent experiments, with means shown. ANOVA and Bonferroni *post hoc* tests were carried out. ns, no significant difference.

Following Rab5 dissociation, phagosomes sequentially acquire late endosome and lysosomal markers. Rab7 is a GTPase associated with late endosomes and is essential in regulating the progression of phagosome maturation (24). To ascertain the kinetics of Rab14 localization to phagosomes containing C. albicans cells in relation to Rab7 kinetics, we first cotransfected RAW 264.7 macrophages with constructs expressing tRFP-Rab7 and eGFP-Rab14 or either of the dominant negative variants and then monitored their localization dynamics during the phagocytosis of C. albicans cells. Live-cell video microscopy showed that in the initial stages of phagosome maturation, during the localization of Rab14 on phagosomes, Rab7 was barely visible on phagosomal membranes. However, after the dissociation of Rab14 from phagosomes, Rab7 became localized to phagosomes and was retained on the phagosomes for prolonged periods, extending beyond several hours (Fig. 7A) (see also Video S4 in the supplemental material). Notably, although Rab7 localization was not significantly different in cells expressing eGFP-Rab14 or eGFP-Rab14^S25N^, macrophages expressing eGFP-Rab14^N124I^ tended to exhibit a slight delay in tRFP-Rab7 localization to phagosomes, taking an average of around 35 min compared to around 25 min (Fig. 7B). To further investigate the relationship between phagosomal Rab14 and Rab7, and specifically to determine whether Rab14 depletion affected Rab7 localization to phagosomes, RAW 264.7 macrophages were first cotransfected with Rab14 siRNA and tRFP-Rab7 and then observed by live-cell imaging. A pattern similar to that with Rab14 dominant negative variants was observed, in that Rab14 depletion did not significantly affect Rab7 localization (Fig. 7C).

### Rab14 affects late-stage phagosome maturation.

Late-stage maturation of phagosomes containing C. albicans cells was investigated using antibodies to LAMP1, a late endosome and lysosome glycoprotein that is specifically localized to acidic lysosome structures (35). Rab14-silenced J774.1 macrophages were incubated with C. albicans cells for 45 min or 3 h prior to LAMP1 immunostaining.

**FIG 8:**
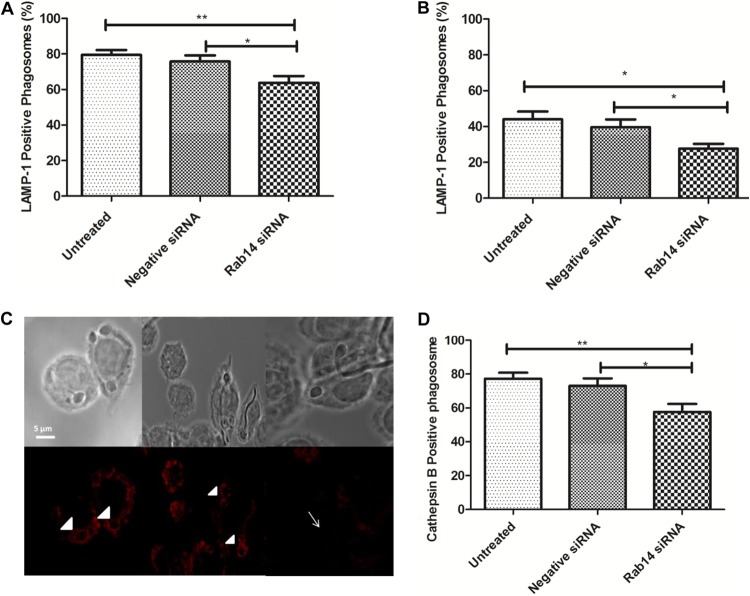
Reduced association of late phagosome maturation markers with phagosomes containing C. albicans cells. (A and B) Percentage of LAMP1-positive phagosomes at 45 min (A) or 3 h (B) after infection of Rab14-depleted J774.1 macrophages with C. albicans cells. Data are means ± SEM for at least three independent experiments. At least 50 phagosomes were analyzed per experiment. (C) Immunostaining of the lysosomal marker LAMP1 with an APC-conjugated anti-LAMP1 antibody in phagosomes of untreated (left), negative-control siRNA-transfected (center), and Rab14 siRNA-transfected (right) macrophages containing live C. albicans cells. Arrowheads and arrows point to LAMP1-positive and LAMP1-negative phagosomes, respectively. (D) Quantification of cathepsin B-positive phagosomes. Control and Rab14 siRNA-transfected macrophages were exposed to C. albicans cells for 60 min at 37°C to allow for initial uptake. At the end of the incubation period, a cathepsin detection reagent was added to the cells, and a further 60 min of the interaction was imaged with differential interference contrast and red fluorescence microscopy, with pictures taken every minute. Data are means ± SEM for at least three independent experiments. At least 50 phagosomes were analyzed per experiment. ANOVA and Bonferroni *post hoc* tests were carried out. *, *P* ≤ 0.05; **, *P* ≤ 0.01.

At 45 min post-C. albicans infection, we observed a significant reduction in the proportion of LAMP1-positive phagosomes in Rab14 siRNA-transfected macrophages (64.82% ± 4.36%) compared to untreated (77.61% ± 2.75%) or negative-control siRNA-transfected (76.11% ± 3.23%) macrophages (Fig. 8A). By 3 h, an overall reduction in LAMP1 localization from that at the 45-min time point was observed. More importantly, the percentage of LAMP1 phagosomes was significantly lower in Rab14 siRNA-transfected macrophages than in negative-control siRNA-transfected macrophages (27.6% ± 2.7% and 39.7% ± 4.3%, respectively [*P* ≤ 0.05]), indicating that the defect in LAMP1 acquisition caused by Rab14 depletion is more pronounced at late stages of phagosome maturation (Fig. 8B and C).

Later stages of phagosome maturation are also characterized by the acquisition of lysosomal cysteine proteases, such as cathepsins. Having observed a change in LAMP1 association with phagosomes, we thought it important to investigate the influence of Rab14 function on lysosomal hydrolytic enzymes. We examined the localization of activated cathepsin B after the internalization of C. albicans cells by macrophages transfected with Rab14 siRNA, and we observed that in cells depleted of Rab14, cathepsin B was activated in only 57.6% ± 4.8% of phagolysosomes, compared to 77.4% ± 3.5% and 73.0% ± 4.4% in untreated and negative-control siRNA-treated macrophages, respectively (Fig. 8D). Thus, Rab14 depletion disrupts cathepsin B activation in macrophages.

### Rab14-depleted macrophages are more susceptible to killing by C. albicans hyphae than untreated macrophages.

The transition to filamentous growth has been strongly implicated in the virulence of C. albicans and the killing of macrophages following phagocytosis (36, 37). Therefore, the role of Rab14 in the interaction between macrophages and C. albicans cells was investigated using our standard live-cell video microscopy killing assay.

**FIG 9:**
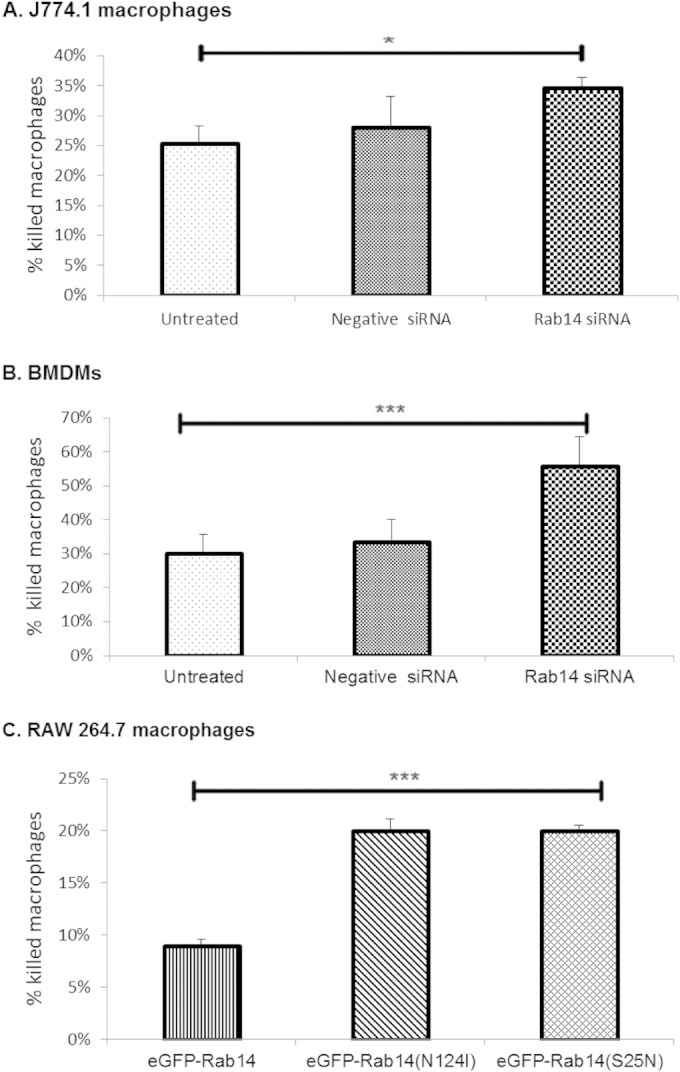
Enhanced killing of Rab14-depleted macrophages by C. albicans. J774.1 macrophages (A), BMDM (B), and RAW 264.7 macrophages (C) depleted of Rab14 by siRNA (A and B) or with expression of dominant negative Rab14 (C) were infected with C. albicans for a 6-h period and were monitored using live-cell microscopy. Graphs represent the percentages of macrophages killed over a 6-h infection period. ANOVA and Bonferroni *post hoc* tests were carried out. *, *P* ≤ 0.05; ***, *P* ≤ 0.001.

Rab14 siRNA-treated macrophages were significantly more susceptible to killing by C. albicans hyphae (35% macrophage lysis by 6 h) than untreated (25% lysis) and negative-control siRNA-treated (28% lysis) macrophages (Fig. 9A). We observed the same pattern of increased macrophage lysis in Rab14 knockdown BMDM and also in RAW 264.7 macrophages transfected with the dominant negative variant eGFP-Rab14^S25N^ or eGFP-Rab14^N124I^ (Fig. 9B and C). The enhanced lysis was fungus mediated and was not due to transfection or knockdown of Rab14 alone (see Fig. S5 in the supplemental material). In addition, the influence of Rab14 on the viability of C. albicans cells was assessed following phagocytosis; however, Rab14 did not detectably alter the potency of macrophages against the fungus (see Fig. S6 in the supplemental material). Thus, Rab14 is active during the early stages of phagosome maturation, affects late-stage phagosome maturation, and protects macrophages against killing by the fungus.

## DISCUSSION

We show here that Rab14 transiently localizes to phagosomes containing live C. albicans cells shortly after engulfment. In contrast to that of Rab5, the duration of Rab14 localization is dependent on fungal morphology within the phagosome and is directly proportional to hyphal size. Furthermore, we found that silencing Rab14 had no effect on markers of early phagosome maturation and did not significantly affect Rab5/Rab7 conversion but delayed the acquisition of key markers of late stages of the maturation process. Importantly, the delay observed in late-stage phagosome maturation was associated with a significant increase in the ability of the pathogen to escape from and kill macrophages after phagocytosis. A schematic diagram of these findings is presented in Fig. S7 in the supplemental material. Therefore, Rab14 activity promotes phagosome maturation during C. albicans infection but is dysregulated on the phagosome in the presence of the invasive hyphal form, which favors fungal survival and escape.

Phagosome maturation is a complex process in which the nascent phagosome undergoes a series of fission and fusion events before maturing into an acidic, protease-rich phagolysosome—a process essential for the inactivation of microbes (7). Rab14 has been identified on phagosomes during proteomics studies (21, 22) and has been implicated in the subversion of membrane trafficking by several pathogens (29, 30, 38–40). We show that only wild-type Rab14, not inactive variants, localized to phagosomes containing C. albicans cells in a transient and fungal-morphology-dependent manner. Rab14 was retained on phagosomes containing C. albicans hyphal cells three times as long as it was on phagosomes containing C. albicans yeast cells. Although the possibility was not explored in this study, either the rapid expansion of the fungal cell or the altered composition of the germ tube/hyphal cell wall may influence the retention of Rab14 on those phagosomes. Recent work has revealed differential dynamic actin polymerization around macrophage phagosomes containing live C. albicans yeast and hyphal cells, suggesting that fungal morphology influences the properties of phagosomes (18). Studies have shown that Rab14 is present on maturing bacterium-containing phagosomes and blocks the maturation of mycobacterium-containing phagosomes in macrophages by promoting their fusion with early, but not late, endosomes (29). Rab14 is transiently associated with mycobacterium-containing phagosomes and is maintained for prolonged periods, up to 30 min postinfection (29). Similarly, it has been reported that some Rab14 association was observed around Chlamydia-modified phagosomal inclusions at later stages of infection (38). Thus, some degree of similarity exists between the retention of Rab14 on phagosomes containing hyphal fungal particles, shown here, and on phagosomes containing bacteria that manipulate phagosome maturation for their survival (29, 38).

Rab GTPases have not been directly implicated in the mechanism by which macrophages migrate toward or engulf phagocytic particles, although Rab35 has a role in directing actin bundling during filopodium formation in other cells, which may be required during cell migration (41). We show here that macrophage migration in response to C. albicans cells was independent of Rab14. We also show that the engulfment of C. albicans yeast and hyphal cells was not influenced by Rab14, in contrast to other studies indicating a role for a Rab14-related protein, RabD, in regulating the phagocytic uptake of latex beads by Dictyostelium discoideum (28). A recent study has suggested that the uptake of parasites by macrophages enhances Rab14 expression, which blocks subsequent phagocytic uptake and, ultimately, elimination by macrophages (42). This suggests that different pathogens differ in the way they manipulate their uptake by immune cells. Also, while we used a detailed live-cell imaging system in these studies, other studies were dependent on fixed-time-point analyses measuring phagocytic uptake using spectrofluorometry and confocal microscopy.

Other studies have suggested that viable C. albicans cells interfere with the phagosome maturation process (17, 18, 43), although the implication of Rab14 in this process has hitherto involved nonfungal pathogens (29, 30, 38–40). We observed that Rab14 depletion did not interfere with either LTR localization or Rab5 association with phagosomes containing C. albicans cells. Furthermore, depletion of Rab14 by siRNA in macrophages did not affect the expression of the closely related Rab2 or Rab4 GTPases, suggesting that the effects observed following Rab14 depletion are Rab14 specific. However, the impact of Rab14 depletion on Rab2 and Rab4 activation remains to be further elucidated.

Studies have indicated that following active Rab5 dissociation from a phagosome, Rab7 appears on the phagosome and resides on the membrane during maturation (44). It has been suggested that Rab7 functions downstream of Rab14 in cell corpse phagosomes and that Rab14 is required for the efficient recruitment of Rab7 to the bacterial phagosome (39, 45). We observed that Rab14 depletion did not markedly affect the recruitment of Rab7 to phagosomes containing C. albicans cells. This implies that although Rab14 is present during the Rab5-to-Rab7 transition, it does not interfere with Rab7 function. In contrast with our results, a study with Drosophila hemocytes showed that Rab14 localizes to both Rab5 and Rab7 compartments and regulates Rab7 recruitment during phagosome maturation (45). A reason for this discrepancy could be differences between Drosophila hemocytes and mammalian phagocytes.

We report here that a reduction in Rab14 levels caused a significant decrease in the association of LAMP1 with phagosomes containing C. albicans cells. A detailed study suggested that following the uptake of live C. albicans cells, late endosomal/lysosomal features, including LAMP1 and the lysosomal protease cathepsin D, were lost during phagosome maturation (17). In agreement with this study, we found that at a similar late time point postinfection (3 h), fewer phagosomes containing C. albicans cells were LAMP1 positive than at an earlier time point (45 min), perhaps suggesting that phagosomal proteins had cycled out. Nevertheless, the depletion of Rab14 at both time points investigated in this work was associated with reduced LAMP1 acquisition by phagosomes.

We also demonstrate that the level of recruitment of cathepsin B by phagosomes containing C. albicans cells was reduced following Rab14 knockdown. Cathepsin has optimal activity within the acidic and reducing environment of lysosomes (46). We show that LTR staining was unaffected by Rab14 knockdown, but our data suggest that even though initial acidification is not affected, it is likely that the phagosomes containing C. albicans cells do not fully mature and acidify, thus limiting cathepsin activation. Rab7 and LAMP1 are both markers of late endosomes (24). However, studies using two-color live-cell imaging identified three distinct populations of endolysosomal vesicles: Rab7-positive vesicles, LAMP1-positive vesicles, and vesicles positive for both Rab7 and LAMP1 (47, 48). Therefore, Rab14 may specifically regulate the fusion of distinct LAMP1-positive late endosomes with phagosomes containing C. albicans cells. LAMP1 also marks lysosomes, so its depletion in Rab14 knockdown cells may be indicative of disrupted fusion of lysosomes to phagosomes. This interpretation is further supported by the diminished cathepsin levels within phagosomes containing C. albicans cells, since these proteases originate specifically from lysosomal compartments (49).

We also show that reducing Rab14 expression in macrophage cell lines and primary macrophages enhanced susceptibility to killing by C. albicans hyphal cells. We suggest that this was due to a defect in lysosome fusion and cathepsin activation, which ultimately disrupted the biogenesis of C. albicans-containing phagolysosomes with full degradative qualities. This contradicts data from studies of bacterial phagosomes where Rab14 RNA interference released the maturation block and allowed phagosomes harboring live mycobacteria to progress into phagolysosomes (29). This discrepancy suggests that these two pathogens have evolved different strategies for survival within macrophage phagosomes. Mycobacteria inhibit phagosome maturation and survive within an immature phagosome, whereas C. albicans develops hyphae and escapes (37, 50, 51). The role of Rab14 in phagosome maturation during the processing of other filamentous fungi remains to be examined.

In conclusion, our study demonstrates that Rab14 is temporarily associated with phagosomes and that the temporal regulation of recruitment is markedly influenced by C. albicans morphology and is directly proportional to hyphal length. Rab14 association occurred at a stage partially overlapping Rab5 association and upstream of Rab7 function. Rab14 affects the maturation of phagosomes containing C. albicans cells, resulting in impaired fusion of lysosomes with the phagosome and ultimately in protection against macrophage killing by hypha-mediated lysis. Future work will address the mechanistic strategies used by this pathogen to avoid innate immune mechanisms.

## Supplementary Material

Supplemental material
