# Supplementary material for: Rab14 Regulates Maturation of Macrophage Phagosomes Containing the Fungal Pathogen Candida albicans and Outcome of the Host-Pathogen Interaction
Source: Infect Immun. 2015 Mar 17;83(4):1523–35. doi: 10.1128/IAI.02917-14 (PMC4363425; doi:10.1128/IAI.02917-14)
Supplement: Supplemental material [file supp_83_4_1523__index.html]

Rab14 Regulates Maturation of Macrophage Phagosomes Containing the Fungal Pathogen Candida albicans and Outcome of the Host-Pathogen Interaction — Supplemental material 

# Rab14 Regulates Maturation of Macrophage Phagosomes Containing the Fungal Pathogen Candida albicans and Outcome of the Host-Pathogen Interaction

## Supplemental material

**Files in this Data Supplement:**

- Supplemental file 1 -

  Fig. S1. eGFP-Rab14 and anti-Rab14 staining in RAW264.7 cells expressing *eGFP-Rab14* or *eGFP-Rab14N124I*. Fig. S2. Anti-Rab14-stained BMDM following phagocytosis of *C. albicans*. Fig. S3. Knockdown of Rab14 in J774.1 macrophages using siRNA, shown as Western blot and qPCR data. Fig. S4. Knockdown of Rab14 using siRNA in RAW264.7 macrophages transfected with GFP-Rab5, shown as Western blot and qPCR data. Fig. S5. Survival of *C. albicans* recovered from phagosomes in Rab14 knockdown or control RAW264.7 macrophages. Fig. S6. Survival of *C. albicans* recovered from phagosomes in Rab14 knockdown or control RAW264.7 macrophages. Fig. S7. Schematic diagram of the temporal localization of Rab14 on phagosomes containing *C. albicans* and the consequences of disrupting Rab14 upon phagosome maturation. Legends for Videos S1 to S4.

  PDF, 363K
- Supplemental file 2 -

  Video S1. RAW264.7 macrophages expressing eGFP-Rab14 phagocytosing live *C. albicans* yeast.

  AVI, 1.5M
- Supplemental file 3 -

  Video S2. RAW264.7 macrophages expressing eGFP-Rab14 phagocytosing live *C. albicans* hyphae.

  AVI, 1.2M
- Supplemental file 4 -

  Video S3. RAW264.7 macrophages expressing eGFP-Rab14*S25N* phagocytosing *C. albicans*.

  AVI, 1.0M
- Supplemental file 5 -

  Video S4. RAW264.7 macrophages cotransfected with eGFP-Rab14 and tRFP-Rab7 phagocytosing *C. albicans*.

  AVI, 1.6M
