# Supplementary material for: Rab14 Regulates Maturation of Macrophage Phagosomes Containing the Fungal Pathogen Candida albicans and Outcome of the Host-Pathogen Interaction
Source: Infect Immun. 2015 Mar 17;83(4):1523–35. doi: 10.1128/IAI.02917-14 (PMC4363425; doi:10.1128/IAI.02917-14)
Supplement: Supplemental material [file IAI.02917-14_zii999091159so1.pdf]

Supplemental Data

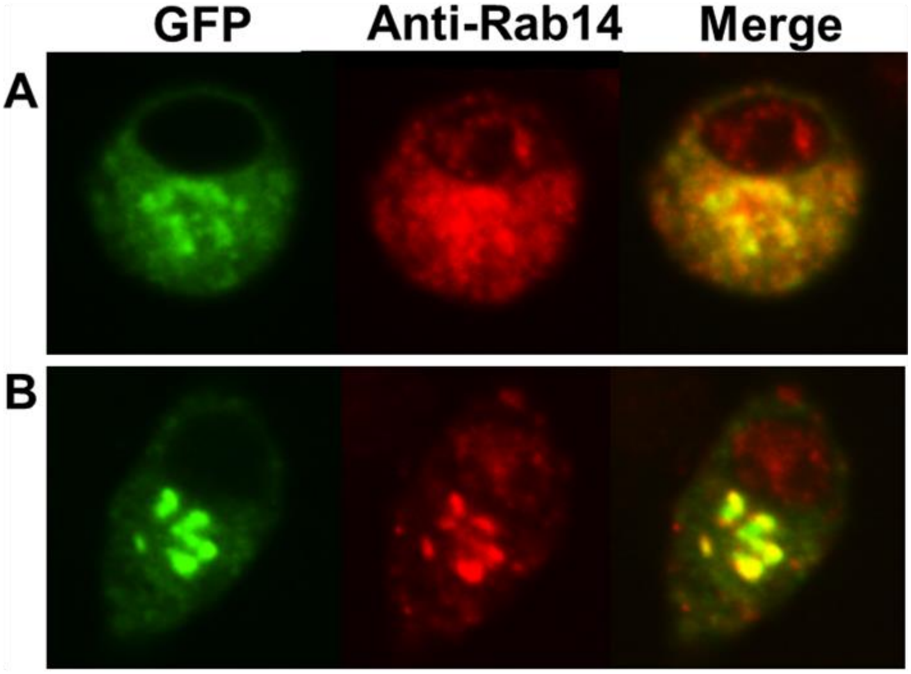

Figure S1: eGFP-Rab14 and Anti-Rab14 staining in RAW264.7 expressing A – *eGFP-Rab14* or B - *eGFP-Rab14*^{N124I}

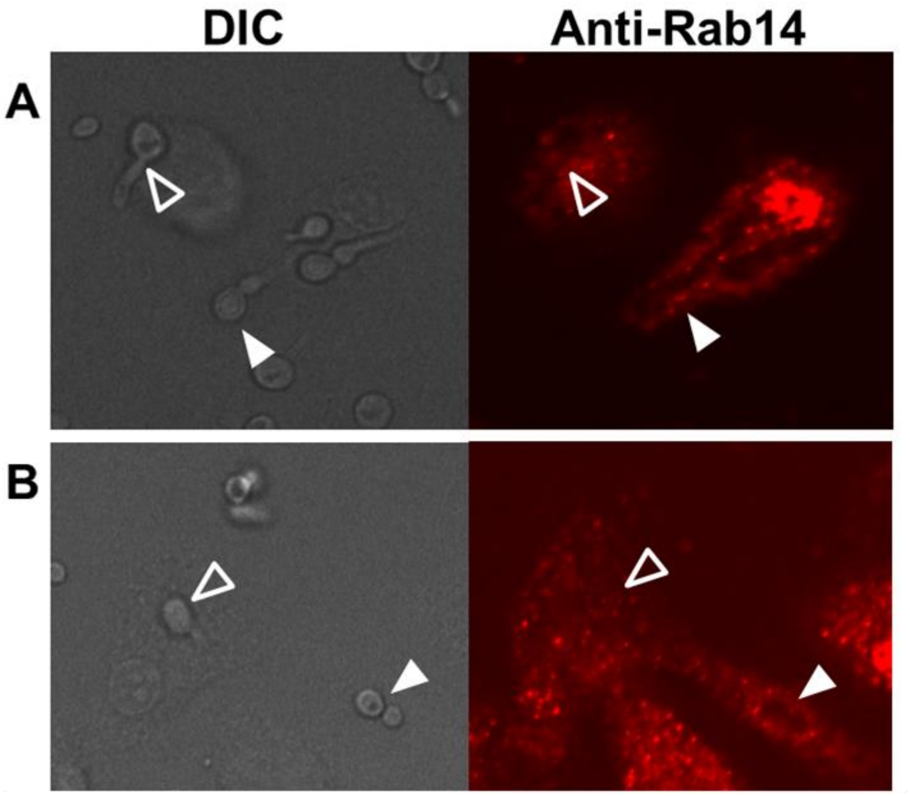

Figure S2: Anti-Rab14 stained BMDM following phagocytosis of *C. albicans*. Enhanced Rab14 localisation around some phagosomes (closed arrows) but not others (open arrow) within two different cells (A and B).

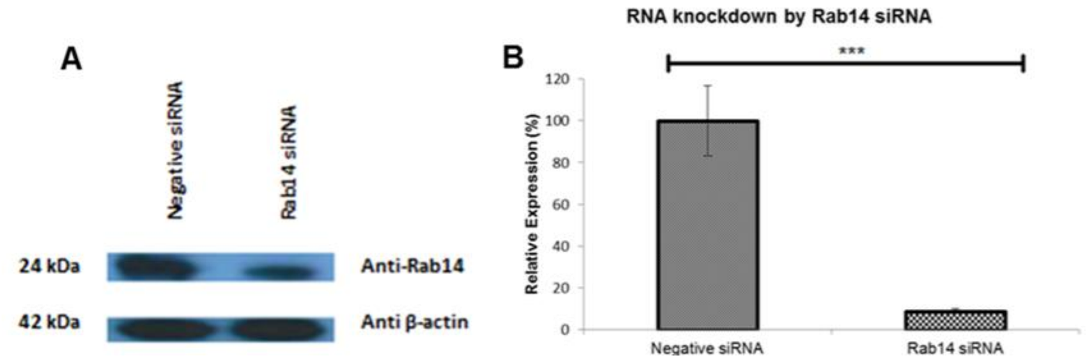

Figure S3: Knockdown of Rab14 in J774.1 macrophages using siRNA shown as A – western blot and B – qPCR data.

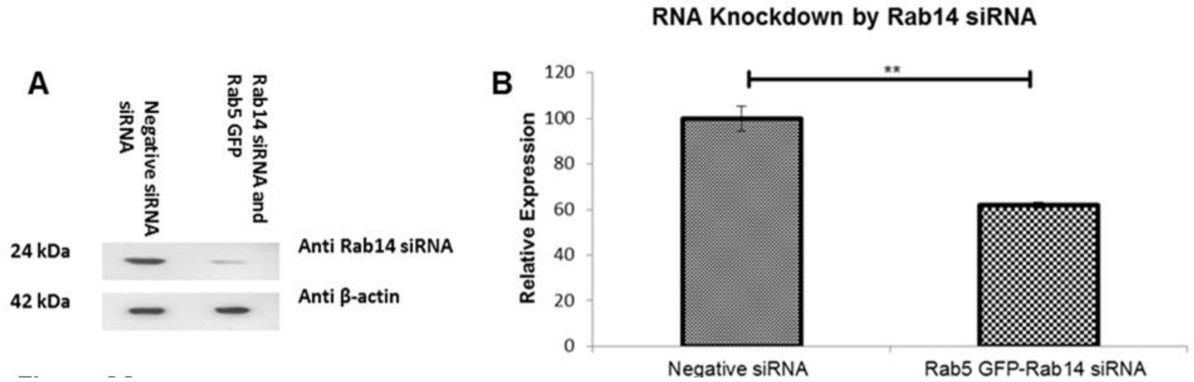

Figure S4: Knockdown of Rab14 using siRNA in RAW264.7 macrophages transfected with GFP-Rab5 shown as A – western blot and B – qPCR data.

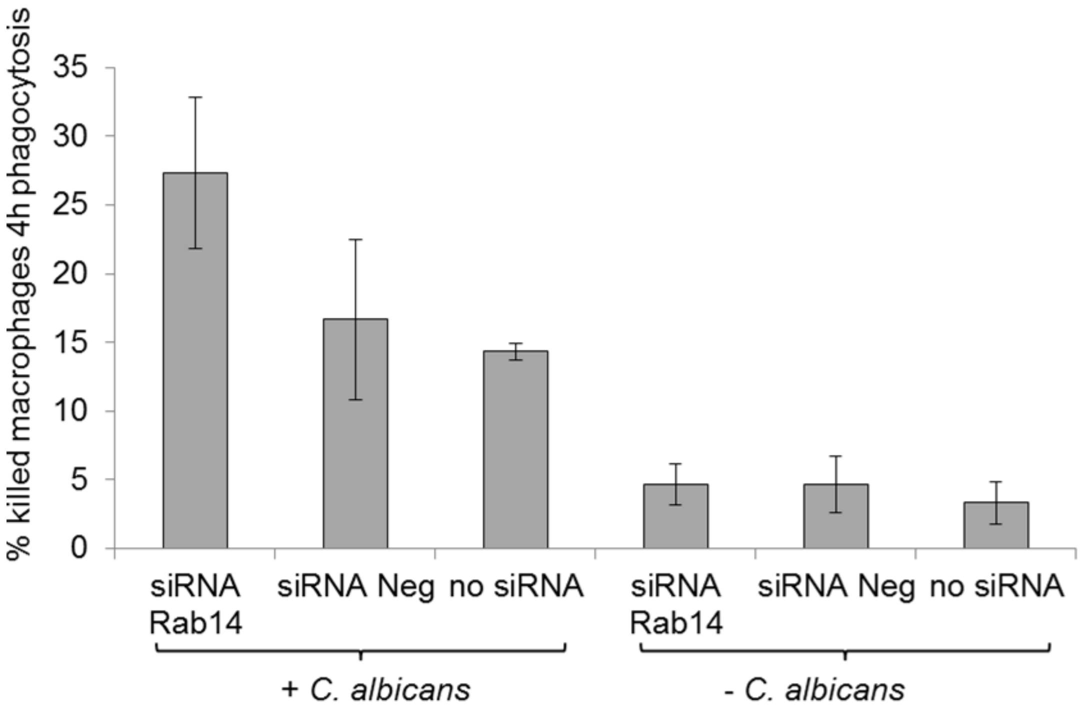

Figure S5: Survival of *C. albicans* recovered from phagosomes in Rab14 knockdown or control RAW264.7 macrophages. Fungi were liberated from phagocytosis assays following 4 h incubation with macrophages.

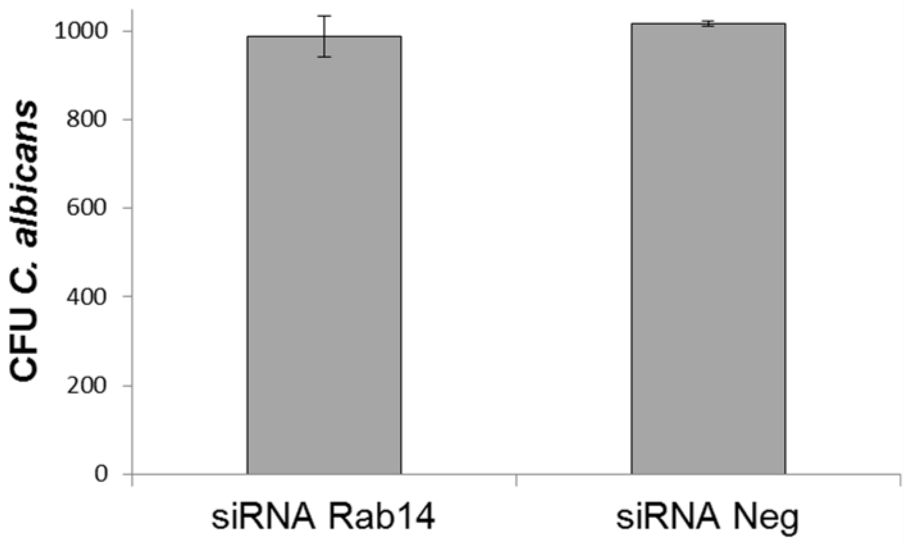

Figure S6: Survival of *C. albicans* recovered from phagosomes in Rab14 knockdown or control RAW264.7 macrophages. Fungi were liberated from phagocytosis assays following 4 h incubation with macrophages.

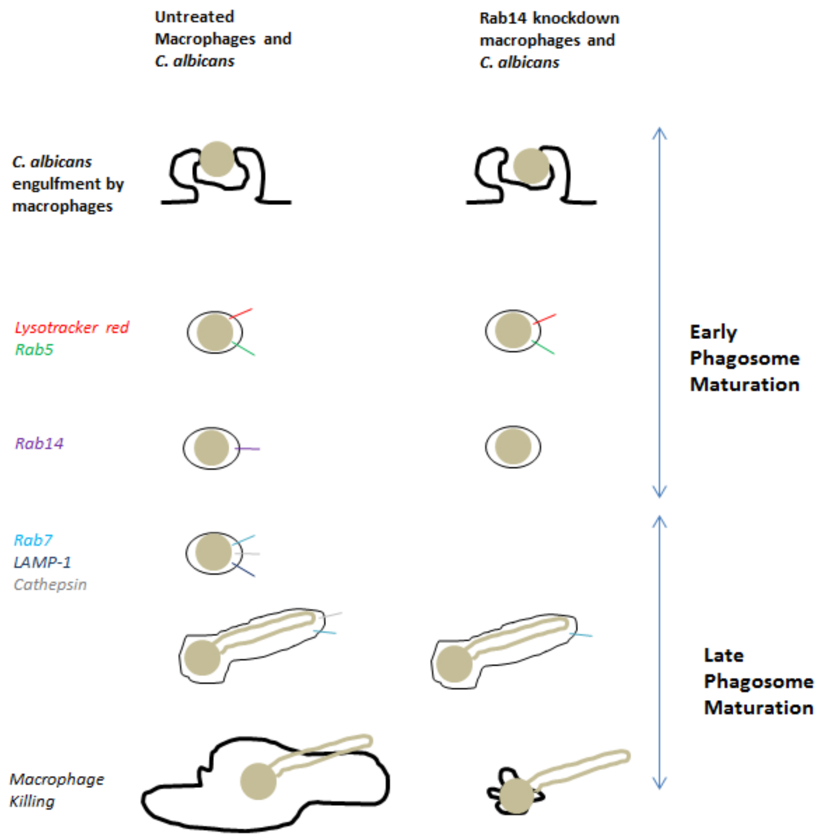

Figure S7: Schematic diagram of the temporal localisation of Rab14 on phagosomes containing *C. albicans* and the consequences of disrupting Rab14 upon phagosome maturation. . (1) C. albicans engulfment by macrophages. (2) In wild-type and Rab14 deficient cells, Rab5 is recruited and interacts with both yeast and hyphal *C. albicans*. Comparable lysotracker red staining between control and Rab14 knockdown cells. Rab14 association to phagosomes containing *C. albicans* commences and it partially colocalises with Rab5. In Rab14 deficient cells (expressing dominant negative Rab14), there is no Rab14 association to the phagosomes. (3) Rab14 is retained on hyphal *C. albicans* for longer than it does on the yeast form. (4) Rab14 dissociates from phagosomes and this is followed by Rab7 association which is not affected in the absence of Rab14. LAMP1 expression is lost on Rab14 deficient phagosomes containing yeast and hyphal *C. albicans* forms compared to untreated control macrophages which acquire LAMP1. There is defective cathepsin B activation in Rab14 deficient phagosomes. In control macrophages, acquisition of LAMP1, in addition to cathepsin activity, are required for phagosomes containing *C. albicans* to mature into phagolysosomes contributing to the protection against fungal mediated lysis of host cells. (5) Phagosomes from Rab14 knockdown cells are defective for LAMP1 and active cathepsin acquisition,

and thereby exert less control upon internalised *C. albicans* which ultimately kill the macrophages.

Video S1: RAW264.7 macrophages expressing eGFP-Rab14 phagocytosing live *C. albicans* yeast

Video S2: RAW264.7 macrophages expressing eGFP-Rab14 phagocytosing live *C. albicans* hyphae

Video S3: RAW 264.7 macrophages transfected with eGFP-Rab14^{S25N} phagocytosing *C. albicans*.

Video S4: RAW 264.7 macrophages co-transfected with eGFP-Rab14 and tRFP-Rab7 phagocytosing *C. albicans*.
